# Development and Validation of a Concise Objectifiable Risk Evaluation Score for Non-Relapse Mortality after Allogeneic Hematopoietic Stem Cell Transplantation

**DOI:** 10.3390/cancers16030515

**Published:** 2024-01-25

**Authors:** Gunnar Weise, Radwan Massoud, Rolf Krause, Silke Heidenreich, Dietlinde Janson, Evgeny Klyuchnikov, Christine Wolschke, Gaby Zeck, Nicolaus Kröger, Francis Ayuk

**Affiliations:** Department of Stem Cell Transplantation, University Medical Center Hamburg-Eppendorf, Martinistr. 52, 20251 Hamburg, Germany; weise.gunnar@gmail.com (G.W.); radwan.massoud@uke.de (R.M.); r.krause@uke.de (R.K.); s.heidenreich@uke.de (S.H.); d.janson@uke.de (D.J.); e.klyuchnikov@uke.de (E.K.); wolschke@uke.de (C.W.); g.zeck@uke.de (G.Z.); nkroeger@uke.de (N.K.)

**Keywords:** allogeneic hematopoietic stem cell transplantation, non-relapse mortality, overall survival, objectifiable risk evaluation, CORE HCT score, HCT comorbidity index

## Abstract

**Simple Summary:**

This study aimed to create a simple and reliable tool, the CORE HCT score, to predict the chances of non-relapse mortality (NRM) and overall survival (OS) after allogeneic hematopoietic stem cell transplantation (allo-HCT). Using data from 1120 adult patients who had undergone this procedure at our center between 2013 and 2020, we identified specific patient factors affecting NRM: serum albumin, serum creatinine, serum C-reactive protein, heart and lung function, and age. Factors were weighted according to their impact on NRM. The resulting CORE HCT score grouped patients into low-, medium-, and high-risk categories, showing its effectiveness across different conditions and donor types. Notably, compared with the HCT Comorbidity Index (HCT-CI), the CORE HCT score performed better in predicting NRM and OS. The findings were validated in two independent cohorts, which supports the utility of the CORE HCT score in guiding risk assessment for allo-HCT in adult patients with malignant diseases.

**Abstract:**

We aimed to develop a concise objectifiable risk evaluation (CORE) tool for predicting non-relapse mortality (NRM) and overall survival (OS) after allogeneic hematopoietic stem cell transplantation (allo-HCT). A total of 1120 adult patients who had undergone allo-HCT at our center between 2013 and 2020 were divided into training, first, and second validation cohorts. Objectifiable, patient-related factors impacting NRM in univariate and multivariate analyses were: serum albumin, serum creatinine, serum C-reactive protein (CRP), heart function (LVEF), lung function (VC, FEV1), and patient age. Hazard ratios were assigned points (0–3) based on their impact on NRM and summed to the individual CORE HCT score. The CORE HCT score stratified patients into three distinct low-, intermediate-, and high-risk groups with two-year NRM rates of 9%, 22%, and 46%, respectively, and OS rates of 73%, 55%, and 35%, respectively (*p* < 0.001). These findings were confirmed in a first and a second recently treated validation cohort. Importantly, the CORE HCT score remained informative across various conditioning intensities, disease-specific subgroups, and donor types, but did not impact relapse incidence. A comparison of CORE HCT vs. HCT Comorbidity Index (HCT-CI) in the second validation cohort revealed better performance of the CORE HCT score with c-statistics for NRM and OS of 0.666 (SE 0.05, *p* = 0.001) and 0.675 (SE 0.039, *p* < 0.001) vs. 0.431 (SE 0.057, *p* = 0.223) and 0.535 (SE 0.042, *p* = 0.411), respectively. The CORE HCT score is a concise and objectifiable risk evaluation tool for adult patients undergoing allo-HCT for malignant disease. External multicenter validation is underway.

## 1. Introduction

Allogeneic hematopoietic stem cell transplantation (allo-HCT) is a potentially curative treatment option for several malignant and non-malignant hematologic diseases. Increasing availability of donors and procedural improvements, including immunosuppression, prophylaxis, and treatment of infections, have contributed to the improved outcome of this treatment modality over the last few decades [1,2,3,4].

The introduction of reduced-intensity conditioning (RIC) and non-myeloablative conditioning regimens have made transplantation of older, frail, and more severely ill patients feasible [5,6,7,8]. While allo-HCT reduces the risk of relapse of underlying malignancy compared with other conventional therapies, it is associated with a significant risk of toxicity and non-relapse mortality (NRM), which may outweigh the benefits [9]. Therefore, the decision whether to recommend HCT or not requires careful consideration of those factors. Relapse risk is mainly driven by disease biology and remission status at transplant, while NRM is strongly driven by patient fitness and comorbidities [10,11]. Over the past few decades, several scores have been developed for the prediction of NRM risk taking into account patient-related, disease-related, or combined parameters [12,13,14,15,16,17,18], the HCT Comorbidity Index (HCT-CI) being the most commonly used [13].

The HCT-CI, which builds on the Charlson Comorbidity Index (CCI), further defines transplant-specific risks that can be partially objectified, with the goal of predicting two-year NRM. Three risk groups were identified by the HCT-CI score: 0 points, low risk; 1–2 points, intermediate risk; and 3 points, high risk. The corresponding two-year NRM rates are 14%, 21%, and 41%, respectively [13]. The HCT-CI has been repeatedly adjusted by adding parameters such as age with a cut-off at 40 years (HCT-CI/age), ferritin, albumin, and platelets (augmented HCT-CI) or combinations of augmented HCT-CI, age risk groups, as well as cytogenetic and molecular risk groups (AML composite model, AML-CM) [19,20,21]. Although being well established in clinical practice, the HCT-CI is limited by subjective user interpretation and the need for a detailed, sometimes patient-reported, medical history. Furthermore, the 40 years cut-off of the HCT-CI/age is not representative of current transplant practice, where the median patient age became much higher [22,23]. Other widely used scores do not focus on comorbidities: the EBMT score includes patient and donor factors, the endothelial activation and stress index (EASIX) uses a formula reflecting endothelial dysfunction, while the Dana–Farber Cancer Institute (DFCI) score focuses on disease risk factors [14,15,17].

This study set out to identify objectifiable patient-related, comorbidity-associated parameters that impact the risk of NRM and use them to create a concise tool for the prediction of patient outcome after allo-HCT.

## 2. Methods

### 2.1. Patients

Consecutive adult patients (≥18 years old) with malignant hematological diseases (AML, ALL, CML, MDS, MPN, multiple myeloma, or lymphomas, (Table 1)) who underwent an allo-HCT between 2013 and 2020 were included; all other patients were excluded. GVHD prophylaxis included cyclosporine A and mycophenolic acid in 97% and ATG in 81% of the patients. A pre-specified data set of measurable patients’ characteristics prior allo-HCT that may reflect comorbidities was collected for every patient and included disease, age, laboratory tests, lung function tests, and echocardiography results (Supplementary Table S1). The timepoint for data collection was marked by the date of hospital admission for allo-HCT. In case of prior hospitalization of the patient, results from the last check-up prior to the start of conditioning were used. Overall, 915 patients fulfilled the criteria and were included. Next, patients transplanted between 2013 and 2018 were randomly assigned to a training cohort (617 patients) to construct the score and a first validation cohort (298 patients) to test for consistency. The Chi-squared test was used to check for independency of the two cohorts. Only patients with complete laboratory data and functional parameters were eligible for multivariate analysis. For a second validation, all patients who underwent allo-HCT between 2019 and 2020 (*n* = 205) were included. Data were retrieved from our clinical database and data sets were completed with data extracted from patients’ electronic files.

### 2.2. Statistical Analysis

Whenever possible, the Common Terminology Criteria for Adverse Events, CTCAE (National Cancer Institute, November 2017, CTCAE v5.0, www.ctep.cancer.gov, last viewed 19 November 2022 19:00), was used to classify the laboratory and functional parameters into categorized factors. For continuous variables, the median was calculated to obtain a categorizable factor. Due to clinical relevance, the factor age was subdivided into three categories: under 60, between 60 and 69, and over 70. Non-relapse mortality and relapse were assessed using Fine and Gray’s test for competing risks, with relapse as a competing event for death from other causes. The Cox regression model and the Kaplan–Meier method were used to calculate overall survival (OS). NRM was defined as death without relapse or progression of underlying disease. The results are presented with concordant estimated hazard ratios (HR) and 95% confidence intervals on both sides. All parameters in patient demographics were included in the univariate analysis. Parameters with *p* < 0.1 were entered into a Cox regression multivariate model with stepwise backward elimination. The area under the receiver operating characteristic (ROC) was assessed, and the findings are presented in terms of c-statistics with the corresponding standard error (SE). All reported *p*-values are two-sided, and *p*-values < 0.05 were considered statistically significant. Statistical analysis was performed using SPSS (version 27) and RStudio (version 1.3.1073).

### 2.3. Score Development

First, a univariate analysis for NRM was performed on the training cohort (*n* = 617), followed by multivariate analysis including all parameters with *p* < 0.1 in univariate analysis. All parameters were adjusted for disease risk in accordance with Sorror et al. 2005 [13], whereby leukemia in first remission, CML in first chronic phase, and MDS-refractory anemia were defined as low-risk diseases, while all other diseases were classified as high risk. The obtained hazard ratios were weighted, with 1 point for HR 1.2–2.0; 2 points for HR 2.1–3.0, and 3 points for HR > 3.0. Categories of parameters that yielded only a few patients and did not contribute to the risk differentiation because of inconsistent HRs were merged with the corresponding higher category. This was the case for the left ventricular ejection fraction (LVEF) < 20% and serum creatinine < 3–6xULN (Table 1 and Table 2). The calculated score values were allocated to 3 respective risk groups—low risk, intermediate risk, and high risk. The constructed score was then tested in a first validation cohort (*n* = 298) and a second (more recent) validation cohort (*n* = 205) to check for consistency.

## 3. Results

In the training cohort, 119 patients experienced the event of NRM, while 167 patients had relapse of underlying malignancy. The median survival time for the entire cohort was 1673 days. Death occurred in 234 patients, and the median survival time among the patients who died was 154 days.

### 3.1. Patient-Specific Risk Score

The univariate analysis included more than 20 patient-related parameters (Supplementary Table S1). Factors with *p* < 0.1 in univariate analysis were then entered into the multivariate model and weighted according to the associated hazard ratios, as described in the methods section (Table 1 and Table 2). Factors that did not impact NRM risk were removed through stepwise backward elimination. This was the case for the factors NT-proBNP and CMV-status. Accordingly, serum albumin < 20 g/L was assigned a weighted score of 3. Serum creatinine > 1.5xULN and age above 70 years were assigned a score of 2. A score of 1 was assigned to serum albumin < LLN–20 g/L, serum creatinine > ULN–1.5xULN, left ventricular ejection fraction (LVEF) ≤ 50%, forced expiratory volume (FEV1) < 60%, vital capacity (VC) < 75%, C-reactive protein (CRP) ≥ 6 mg/L, and age 60–69 years.

The sum of the weighted scores assigned to each patient in the training cohort ranged from 0 to 9 points, with rising scores indicating a higher NRM risk. Patients with a score of 0 or 1 points had the lowest two-year (2yr) NRM rates at 12% and 7%, respectively. Forty percent of the patients in the training set could be assigned to score 0 or 1. Patients who achieved two and three points had 2yr NRM rates of 22% and 19%, respectively. They accounted for 25% and 18% of the patients in the training set, respectively. Nine percent of patients from the training cohort had four points or at least five points. With four points, a two-year NRM of 28% was seen, whereas five or more points was associated with the highest NRM of 46% (Supplementary Table S2).

**Figure 1:**
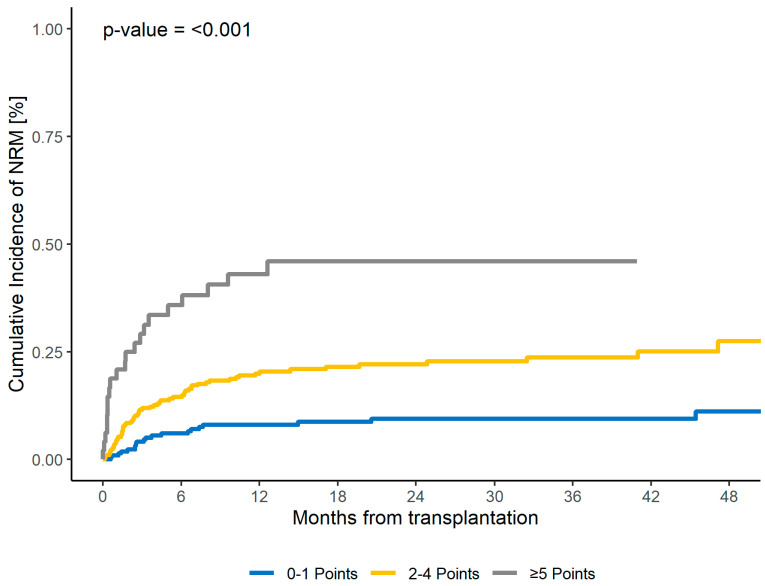
Two-year NRM for CORE score for the training cohort.

Three risk groups were identified based on the sum of points, suggesting a possible three-point risk score (CORE HCT) that differentiates the risk groups as follows: low risk with zero or one point, intermediate risk with two, three, or four points, and high risk with at least five points. The corresponding NRM rates were 9% for the low-risk group, 22% for the intermediate-risk group, and 46% for the high-risk group. The CORE HCT risk score was predictive for 2yr NRM both in the training cohort (*p* < 0.001) and in the first validation cohort (*p* < 0.001) (Table 3 and Table 4, Figure 1, Supplementary Figure S1). Subsequently, the c-statistics for NRM were evaluated for the first validation cohort, revealing a value of 0.63 (SE 0.04, *p* = 0.003).

### 3.2. Survival and Relapse

**Figure 2:**
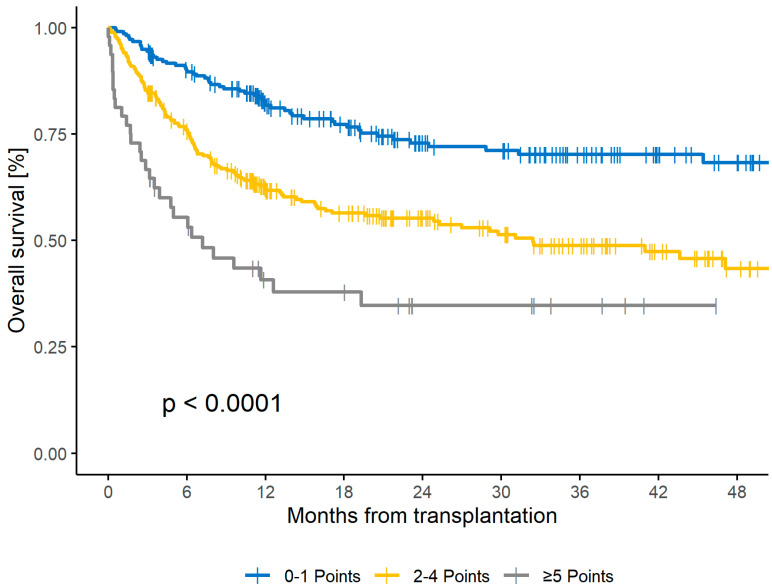
Two-Year OS for CORE score for the training cohort.

Accordingly, the CORE HCT score was also predictive for OS. The corresponding 2yr OS rates for low-, intermediate-, and high-risk scores were 73%, 55%, and 35%, respectively (*p* < 0.001), in the training set and 73%, 47%, and 18%, respectively (*p* < 0.001), in the first validation set (Figure 2 and Supplementary Figure S2). The C-statistics for OS in the first validation cohort were 0.659 (SE 0.03, *p* < 0.001). Regarding relapse rates, no significant influence (*p* = 0.243) was seen in the training cohort. For the low-, intermediate-, and high-risk groups, the two-year relapse incidences were 27%, 31%, and 16%, respectively.

### 3.3. Second Validation

Patients in the 2013–2018 cohort were randomly assigned to a training cohort to construct the score and a first validation cohort to check for consistency. For further validation, a more recently treated cohort of 205 patients who underwent allo-HCT between 2019 and 2020 was analyzed. In this group, NRM was seen in 33 cases, while 52 patients experienced disease relapse. The cohort had a median survival time of 904 days. There were 71 cases of mortality, with the median survival time for individuals who experienced death being 174 days. The CORE HCT score was also predictive for two-year NRM and two-year OS in this second validation cohort, with *p* = 0.002 and *p* < 0.001, respectively (Table 5, Supplementary Table S3, and Supplementary Figures S3 and S4). The c-statistics in this cohort for NRM and OS were 0.666 (SE 0.05, *p* = 0.001) and 0.675 (SE 0.039, *p* < 0.001), respectively.

Next, for comparison, the impact of HCT-CI on NRM was analyzed in this patient cohort. The distribution of risk groups indicated that 13% of patients were classified as low risk, 26% as intermediate risk, and 61% as high risk. The corresponding two-year NRM rates for these groups were 30%, 16%, and 16%, respectively (*p* = 0.249), and the two-year OS rates were 64%, 73%, and 60%, respectively (*p* = 0.29). The C-statistics for NRM and OS were 0.431 (SE 0.057, *p* = 0.223) and 0.535 (SE 0.042, *p* = 0.411), respectively.

### 3.4. CORE Score Subgroup Analysis

After demonstrating the ability of the CORE HCT score to predict NRM and OS outcome in all three cohorts, analysis of the subgroups was performed. For this, patients with complete data sets from the training and the two validation cohorts were included (*n* = 1025). The CORE HCT score predicted NRM in patients who received myeloablative conditioning (MAC, *n* = 583, *p* < 0.001), reduced-intensity conditioning (RIC, *n* = 442, *p* < 0.001), had received ATG (*n* = 828, *p* < 0.001), had conditioning without ATG (*n* = 197, *p* < 0.001), with underlying disease AML (*n* = 397, *p* < 0.001), MDS (*n* = 150, *p* = 0.027), myelofibrosis (*n*= 133, *p* = 0.003), had transplants from matched related donors, MRD (*n* = 205, *p* < 0.001), unrelated donors (*n* = 793, *p* < 0.001), and haploidentical related donors (*n* = 27, *p* = 0.046). The results of subgroup analysis are summarized in Supplementary Table S4 and Supplementary Figures S5–S8.

### 3.5. Multivariate Analysis for NRM including Donor- and Transplant-Related Factors

We next studied the impact of the CORE HCT score in a multivariate analysis model including donor- and transplant-related factors and all patients with available data from the entire cohort (*n* = 931). The CORE HCT score maintained high significance, with HR of 2.06 (95% CI: 1.4–3.1, *p* < 0.001) and 5.09 (95% CI: 2.9–8.9, *p* < 0.001) for intermediate and high risk compared with low risk, respectively. Interestingly, Eastern Cooperative Oncology Group (ECOG) 3–4 was independently associated with a higher risk of NRM compared with ECOG 1–2 (HR: 2.05; 95% CI: 1.4–3.1; *p* < 0.001). Other borderline significant parameters included the absence ATG in conditioning (HR: 1.52; 95% CI: 1–2.3; *p* = 0.049), patient CMV seropositive status (HR: 1.41; 95% CI: 1–2; *p* = 0.041), and HLA-mismatched unrelated donor transplant (HR: 1.68; 95% CI: 1–2.8; *p* = 0.044) (Supplementary Tables S5 and S6, Supplementary Figure S9). The C-statistic for the combined score of CORE HCT score and ECOG was 0.675 (SE 0.024, *p* < 0.001). In comparison, the CORE HCT score for all three cohorts showed a similar c-statistic of 0.648 (SE 0.022, *p* < 0.001).

## 4. Discussion

This study identified patient-related, comorbidity-associated factors that are associated with an increased risk of NRM. The factors reflect and quantify the functions of the key organs of the lungs, heart, and kidneys, as well as inflammation and patient age, which can all be easily identified by objectifiable laboratory or functional tests. Both VC and FEV1 were independently significantly associated with worse outcomes, emphasizing a central role of lung function in overall patient fitness. Our study demonstrates that a score consisting of the seven factors serum albumin, serum creatinine, CRP, LVEF, VC, FEV1, and age can predict the NRM and OS of patients undergoing allo-HCT. The score can be calculated in a straightforward manner in clinical routine and relies on concise objectifiable parameters that are well-documented during transplant procedures and thus also available for retrospective analysis.

Although biological fitness, rather than patient age alone, is crucial for treatment decisions, age is still a major influence factor on outcome after allo-HCT [24,25,26,27]. To take into account the increasing age of transplant patients, cut-offs at 60 and 70 years were used in our study, differing from the 40 years cut-off in the HCT-CI score [19,22].

With the proposed three CORE HCT risk groups, most patients can be assigned to the low- (39%) and intermediate- (52%) risk groups, showing distinct NRM rates with 11–13% absolute difference in the training, first, and second validation cohorts. This may present a further advantage compared with the HCT-CI score, in which only a 7% absolute difference (14% vs. 21%) was seen between the low- and intermediate-risk groups [13]. The CORE HCT high-risk group accounted for only 9% of the entire population and identified a small, but further distinct risk population. This reflects current transplant practice, whereby only a small fraction of patients with very high risk of NRM undergo the procedure.

The HCT-CI assigns patients to the high-risk group (28% of patients in the initial cohort) if they have three or more points, which is the case when patients, for example, have a single factor, such as a prior solid tumor requiring therapy or corrected diffusing capacity for carbon monoxide (DLCOc) < 66% [13]. The treatment practice and outcome of solid tumors have evolved since 2005, including less aggressive strategies. Consequently, due to the assumed high risk of NRM, patients or treating physicians could reject allo-HCT as treatment option.

In our analysis of the most recently treated patient cohort, we observed no significant correlation between HCT-CI risk groups and NRM or OS. The HCT-CI high-risk group included a rather high proportion of patients (61%) and exhibited a relatively low 2yr NRM rate (16%). The primary factor contributing to the high-risk category was severe pulmonary impairment, particularly a decline in DLCOc, which was prevalent in 73% of high-risk patients in our cohort. Other studies have identified severe pulmonary impairments as the main factor contributing to high-risk HCT-CI scores [13,28,29,30]. The CORE score also includes two lung function parameters (FEV1 and VC), highlighting the importance of pulmonary fitness. However, DLCO and DLCOc did not show a significant impact on NRM in our cohort (Supplementary Table S7). These findings suggest that either HCT-CI places excessive emphasis on DLCO and/or that DLCO is not the most suitable measure for evaluating pulmonary comorbidity in our patient population.

The multivariate analysis including the CORE HCT score and donor and transplant characteristics showed that ECOG and CORE are independent factors impacting outcome. Other significant factors included absent ATG in conditioning, patient CMV seropositive status, and HLA-mismatched unrelated donor transplants. Owing to its subjective character, we chose not to include ECOG in the CORE score, but consider it complementary. The combination of CORE HCT score and ECOG (Table 6 and Supplementary Figure S9) did not improve discrimination, as evidenced by the similar c-statistics of 0.675 vs. 0.648 for CORE and ECOG vs. CORE alone, respectively.

Our study is limited by weaknesses inherent to its retrospective character. The parameters included in the CORE HCT score are, however, precise, objectifiable, and well-documented in patient files. Another constraint in our study lies in the exclusive reliance on data from a singular transplantation center, with both validation cohorts originating from the same center, although the second validation cohort is enriched with the most recent data. Ongoing efforts are dedicated to external validation with comparison of the CORE HCT score and other well-established risk scores.

Though the c-statistics yielded moderate results (0.648), probably due to the complex interplay of numerous (also patient-independent) parameters influencing the outcomes of NRM and OS, these results are comparable to those of the initial publication of the HCT-CI score (0.661) and perform better than the HCT-CI in the second validation cohort [13].

## 5. Conclusions

In conclusion, the CORE HCT risk score consisting of the seven objectifiable, comorbidity-associated clinical parameters is a robust predictor of the two-year NRM and OS. No statistically significant influence was seen regarding relapse incidence. The calculation of the CORE HCT score is straightforward and reduces the risk of subjective user influence and deficiencies due to uncertainties in patient history.

Further modifications and adjustments for each individual disease, including well-established and disease-specific risk factors and donor and transplant parameters could further improve risk scoring in the future. External multicenter validation including comparison with other existing scores is underway and will be necessary to ascertain the value of the CORE HCT score.

## Figures and Tables

**Table 1:** Univariate analysis of patient-specific risk factors for training and first validation.

Factor	Category	*n* (%) ^1^ Training	*n* (%) ^1^ Validation	*p* Chi^2^ (<0.05) ^2^	*p* NRM (<0.1) ^3^ Training
Patients				NS	-
	Number	617 (67)	298 (33)		
Patient age (median, range)				NS	0.017
	<58	310 (50)	156 (52)		
	≥58	307 (50)	142 (48)		
	Range	18–79 years	18–77 years		
Disease				NS	0.03
	AML	245 (40)	97 (33)		
	MDS	90 (15)	52 (17)		
	NHL + HD	64 (10)	32 (11)		
	ALL + other AL	49 (8)	25 (8)		
	MM + PCL	59 (10)	35 (12)		
	OMF + MDS/MPN + CML	110 (18)	57 (19)		
Disease risk				NS	NS
	Low risk	267 (43)	109 (37)		
	High risk	350 (57)	189 (63)		
Albumin (CTC)				NS	<0.001
	≥LLN	369 (60)	186 (62)		
	<LLN–30 g/L	153 (25)	69 (23)		
	<30–20 g/L	89 (14)	40 (13)		
	<20 g/L	5 (1)	3 (1)		
Creatinine (CTC)				NS	0.003
	≤ULN	532 (86)	253 (85)		
	>ULN–1.5xULN	68 (11)	35 (12)		
	>1.5–3xULN	12 (2)	7 (2)		
	>3–6xULN	4 (1)	1 (0)		
	>6xULN	0 (0)	1 (0)		
Left ventricular ejection fraction (CTC)				NS	0.037
	>50%	541 (91)	272 (94)		
	50–40%	43 (7)	16 (6)		
	39–20%	11 (2)	1 (0)		
	<20%	1 (0)	0 (0)		
Vital capacity (CTC)				NS	0.005
	>90%	248 (44)	126 (46)		
	90–75%	209 (37)	101 (37)		
	75–50%	96 (17)	43 (16)		
	<50%	13 (2)	2 (1)		
FEV1 (CTC)				NS	0.002
	>99%	128 (22)	72 (26)		
	99–70%	343 (60)	157 (56)		
	69–60%	51 (9)	29 (10)		
	59–50%	25 (4)	14 (5)		
	<49%	25 (4)	6 (2)		
CRP (median)				NS	0.005
	<6 mg/L	319 (52)	164 (55)		
	≥6 mg/L	298 (48)	134 (45)		

NS—not significant; ULN—upper limit of normal; LLN—lower limit of normal; CTC—common terminology criteria (for adverse events, CTCAE); AML—Acute myeloid leukemia, MDS—Myelodysplastic syndrome, HD—Hodgkin’s disease, NHL—Non Hodgkin’s disease, ALL—Acute lymphoblastic leukemia, AL—Acute leukemia, MM—Multiple myeloma, PCL—Plasma cell leukemia, OMF—Myelofibrosis, MDS—Myelodysplastic syndrome, MPN—Myeloproliferative disorder, CML—Chronic myeloid leukemia. ^1^ Statistic presented: n (%); ^2^ Chi-squared test of independence; ^3^ Gray’s test for competing risk (NRM is shown).

**Table 2:** Multivariate analysis of 2-y-NRM.

Factor	HR (95% CI)	Weighting
Serum albumin		
Serum albumin ≥ LLN	reference	0
Serum albumin < LLN–30 g/L	1.29 (0.77–2.15)	1
Serum albumin < 30–20 g/L	1.60 (0.91–2.81)	1
Serum albumin < 20 g/L	3.47 (0.42–28.53)	3
Serum creatinine		
Serum creatinine ≤ ULN	reference	0
Serum creatinine > ULN–1.5xULN	1.86 (1.09–3.17)	1
Serum creatinine > 1.5xULN	2.21 (0.66–7.35)	2
Left ventricular ejection fraction (LVEF)		
LVEF > 50%	reference	0
LVEF 50–40%	1.62 (0.77–3.4)	1
LVEF < 40%	1.63 (0.52–5.13)	1
Patient age		
Patient age < 60 years	reference	0
Patient age 60–69 years	1.56 (0.95–2.55)	1
Patient age ≥ 70 years	2.14 (1.23–3.75)	2
Forced expiratory volume (FEV1)		
FEV1 ≥ 60%	reference	0
FEV1 < 60%	1.45 (0.72–2.91)	1
Vital capacity (VC)		
VC ≥ 75%	reference	0
VC < 75%	1.43 (0.79–2.59)	1
C-reactive protein (CRP)		
CRP < 6 mg/L	reference	0
CRP ≥ 6 mg/L	1.25 (0.8–1.96)	1

ULN—upper limit of normal, LLN—lower limit of normal; adjusted for disease risk; for the training cohort with *n* = 553.

**Table 3:** Training: CORE risk score prediction for non-relapse mortality, overall survival, and relapse.

Score	Training Cohort									
	Pat. (%)	NRM			OS			Relapse		
		HR (95% CI)	*p* (%)	2-y (%)	HR (95% CI)	*p* (%)	2-y (%)	HR (95% CI)	*p* (%)	2-y (%)
Total			<0.001			<0.001			0.243	
0–1	39	reference		9	reference		73	reference		27
2–4	52	2.63 (1.6–4.3)	<0.001	22	2.24 (1.6–3.1)	<0.001	55	1.11 (0.8–1.5)	0.51	31
≥5	9	6.75 (3.6–12.6)	<0.001	46	4.19 (2.7–6.6)	<0.001	35	0.6 (0.3–1.2)	0.18	16

For the training cohort *n* = 553.

**Table 4:** First validation: CORE risk score prediction for non-relapse mortality, overall survival, and relapse.

Score	First Validation Cohort									
	Pat. (%)	NRM			OS			Relapse		
		HR (95% CI)	*p* (%)	2-y (%)	HR (95% CI)	*p* (%)	2-y (%)	HR (95% CI)	*p* (%)	2-y (%)
Total			<0.001			<0.001			0.571	
0–1	44	reference		13	reference		73	reference		28
2–4	49	2.03 (1.1–3.8)	0.03	24	2.24 (1.5–3.4)	<0.001	47	1.28 (0.8–2)	0.29	34
≥5	7	5.54 (2.2–13.8)	<0.001	48	5.96 (3.2–11)	<0.001	18	1.07 (0.5–2.5)	0.87	20

For the first validation cohort, *n* = 267.

**Table 5:** Second validation: CORE risk score prediction for non-relapse mortality, overall survival, and relapse.

Score	Second Validation Cohort									
	Pat. (%)	NRM			OS			Relapse		
		HR (95% CI)	*p* (%)	2-y (%)	HR (95% CI)	*p* (%)	2-y (%)	HR (95% CI)	*p* (%)	2-y (%)
Total			0.002			<0.001			0.206	
0–1	42	reference		7	reference		81	reference		25
2–4	50	3.08 (1.3–7.6)	0.014	20	3.01 (1.7–5.4)	<0.001	55	1.61 (0.9–2.9)	0.11	31
≥5	8	6.49 (2.1–19.7)	0.001	43 *	5.87 (2.6–13.1)	<0.001	30 *	1.95 (0.7–5.7)	0.22	31 *

For the second validation cohort, *n* = 205; * data only available for day 717.

**Table 6:** Comparison of CORE HCT score and combination of CORE with ECOG and HCT-CI.

	*n*	c-Statistics (SE)	*p*-Value
NRM			
CORE, first validation cohort	267	0.63 (0.044)	0.003
CORE, second validation cohort	205	0.666 (0.05)	0.001
CORE, all cohorts	1025	0.648 (0.022)	<0.001
CORE and ECOG combined, all cohorts	937	0.675 (0.024)	<0.001
HCT-CI, second validation cohort	205	0.431 (0.057)	0.223
OS			
CORE, first validation cohort	267	0.659 (0.034)	<0.001
CORE, second validation cohort	205	0.675 (0.039)	<0.001
CORE, all cohorts	1025	0.644 (0.018)	<0.001
CORE and ECOG combined, all cohorts	937	0.669 (0.018)	<0.001
HCT-CI, second validation cohort	205	0.535 (0.042)	0.411

ECOG—Eastern Cooperative Oncology Group Performance Status; HCT-CI—HCT Comorbidity Index.

## Data Availability

The datasets generated during and analyzed during the current study are not publicly available due to data privacy protection but are available from the corresponding author on reasonable request.

## Supplementary Materials

The following supporting information can be downloaded at: https://www.mdpi.com/article/10.3390/cancers16030515/s1, Table S1: Patients’ characteristics Data; Table S2: Sum of the weighted scores for 2-year NRM; Table S3: Sum of the weighted scores for second validation for 2-years NRM; Table S4: Subgroup analysis for CORE score of the training, first validation and second validation cohorts; Table S5: Multivariate analysis of CORE, transplant-related parameters and performance status; Table S6: Subgroup analysis for ECOG, NRM for CORE and ECOG combined; Table S7: Univariate analysis of the training (set 1) and first validation (set 2) cohort for all analysed parameters; Figure S1: 2-Year NRM for CORE score for the first validation cohort; Figure S2: 2-Year OS for CORE score for the first validation cohort; Figure S3: 2-Year NRM for CORE score for the second validation cohort; Figure S4: 2-Year OS for CORE score for the second validation cohort; Figure S5: 2-year NRM for subgroup MAC; Figure S6: 2-year NRM for subgroup RIC; Figure S7: 2-year OS for subgroup MAC; Figure S8: 2-year OS for subgroup RIC; Figure S9: 2-year NRM of CORE score and ECOG status combined.
