# Supplementary material for: Development and Validation of a Concise Objectifiable Risk Evaluation Score for Non-Relapse Mortality after Allogeneic Hematopoietic Stem Cell Transplantation
Source: Cancers (Basel). 2024 Jan 25;16(3):515. doi: 10.3390/cancers16030515 (PMC10854706; doi:10.3390/cancers16030515)

Supplementary Data

Tables

Table S1: Patients' characteristics Data

**Data**
Patient-related data
Gender
Karnofsky Index
ECOG
BMI
Age
CMV status
Diagnosis
Disease risk
Laboratory data
Serum albumin
Serum bilirubin
Serum creatinine
Serum GOT
Serum GPT
Serum CRP
Serum NT-proBNP
Serum albumin (electrophoresis)
Serum vitamin D
Echocardiography
Transthoracal echocardiography (left ventricular ejection fraction, LVEF)
Lung function tests
Vital capacity (VC)
Forced expiratory volume (FEV1)
Tiffeneau-Index (FEV1/VC)
Diffusing capacity for carbon monoxide (DLCO)
Diffusing capacity corrected for hemoglobin (DLCOc)

ECOG - Eastern Cooperative Oncology Group Performance Status, BMI - Body mass index, CMV - Cytomegalovirus, GOT - Glutamic-oxaloacetic transaminase, GPT - Glutamate-pyruvate transaminase, CRP – C-reactive protein, NT-proBNP - N-terminal pro–B-type natriuretic peptide

Table S2: Sum of the weighted scores for 2-year NRM

	Training cohort			Validation cohort		
Score	Patients (%)	HR (95% CI)	2-y-NRM (%)	Patients (%)	HR (95% CI)	2-y-NRM (%)
0	18	reference	12	21	reference	12
1	22	0.67 (0.3-1.6)	7	23	1.18 (0.4-3.4)	14
2	25	2.02 (1.0-4.0)	22	27	1.31 (0.5-3.6)	16
3	18	1.90 (0.9-4.0)	19	14	3.66 (1.4-9.7)	34
4	9	3.05 (1.4-6.7)	28	9	3.00 (1.0-9.0)	32
≥5	9	5.52 (2.6-11.6)	46	6	6.07 (2.0-18.3)	48

For the training cohort *n*=553, for the validation cohort *n*=267

Table S3: Sum of the weighted scores for second validation for 2-years NRM

	Second validation cohort		
Score	Patients (%)	HR (95% CI)	2-y-NRM (%)
0	18	1	7
1	24	1.5 (0.3-8)	8
2	25	2.65 (0.6-12.4)	14
3	16	3.79 (0.8-18.1)	20*
4	10	8.13 (1.7-37.9)	35
≥5	8	8.36 (1.8-39.9)	43*

For the second validation cohort *n*=205, *data only available for day 717

Table S4: Subgroup analysis for CORE score of the training, first validation and second validation cohorts

Subgroup	Pat (%)	HR (95% CI)	2-year NRM	*p*-value
MAC	583			<0.001
low risk	273 (47)		10	
intermediate risk	282 (48)	2.45 (1.6-3.9)	21	<0.001
high risk	28 (5)	5.28 (2.5-11)	40	<0.001
RIC	442			<0.001
low risk	146 (33)		12	

Subgroup	Pat (%)	HR (95% CI)	2-year NRM	*p*-value
intermediate risk	243 (55)	2.34 (1.3-4.1)	23	0.03
high risk	53 (12)	6.24 (3.3-11.9)	50	<0.001
no ATG	197			<0.001
low risk	90 (46)		15	
intermediate risk	102 (52)	2.01 (1.1-3.8)	27	0.03
high risk	5 (2)	12.95 (3.4-48.8)	-	<0.001
with ATG	828			<0.001
low risk	329 (40)		9	
intermediate risk	423 (51)	2.64 (1.7-4.0)	21	<0.001
high risk	76 (9)	6.46 (3.9-10.9)	44	<0.001
AML	397			<0.001
low risk	118 (30)		11	
intermediate risk	226 (57)	1.82 (0.9-3.6)	16	0.087
high risk	53 (13)	7.13 (3.5-14.6)	51	<0.001
MDS	150			0.027
low risk	76 (51)		10	
intermediate risk	64 (43)	1.46 (0.6-3.7)	14	0.43
high risk	10 (7)	4.89 (1.6-15.3)	42	0.006
HD+NHL				-
low risk	39 (36)		8	
intermediate risk	69 (61)	-	42	-
high risk	3 (3)	-	0	-
MF	133			0.003
low risk	66 (50)		8	
intermediate risk	62 (47)	4.67 (1.6-13.9)	24	0.005
high risk	5 (4)	9.58 (1.9-49.7)	40	0.007
MRD	205			<0.001
low risk	99 (48)		7	
intermediate risk	99 (48)	4.05 (1.6-10)	21	0.003
high risk	7 (3)	13.05 (2.7-62.3)	-	0.001
MUD	793			<0.001
low risk	307 (39)		11	
intermediate risk	414 (52)	2.2 (1.5-3.3)	22	<0.001
high risk	72 (9)	5.27 (3.2-8.7)	45	<0.001
Haplo-ident	27			0.046
low risk	13 (48)		15	
intermediate risk	12 (44)	1.84 (0.4-7.6)	34	0.4
high risk	2 (7)	8.4 (1.9-36.6)	-	0.004

For the training, first validation and second validation cohorts, *n*=1025

MAC - Myeloablative conditioning, RIC - Reduced intensity conditioning, ATG - Anti-thymocyte globulin, AML - Acute myeloid leukemia, MDS - Myelodysplastic syndrome, HD - Hodgkin's disease, NHL - Non Hodgkin's disease, MF – Myelofibrosis, MRD - Matched related donor, MUD - Matched unrelated donor

Table S5: Multivariate analysis of CORE, transplant-related parameters and performance status

Factor	HR (95% CI)	*p*-value
CORE low	*reference*	
CORE intermediate	2.06 (1.4-3.1)	<0.001
CORE high	5.09 (2.9-8.9)	<0.001
ATG based	*reference*	
without ATG	1.52 (1-2.3)	0.049
no TBI	*reference*	
TBI	1.09 (0.7-1.7)	0.7
MAC	*reference*	
RIC	1.2 (0.8-1.7)	0.32
MRD	*reference*	
MUD	1.19 (0.8-1.9)	0.44
Missmatch	1.68 (1-2.8)	0.044
Recipient CMV serostatus negative	*reference*	
Recipient CMV serostatus positive	1.41 (1-2)	0.041
Recipient Donor Sex other than male/female	*reference*	
Recipient Donor Sex male/female	1.53 (1-2.4)	0.06
ECOG 1 + 2	*reference*	
ECOG 3 + 4	2.05 (1.4-3.1)	<0.001

For the training, first and second validation cohorts with *n*=931
Adjusted for disease risk
ATG - Anti-thymocyte globulin, TBI - Total body irradiation, MAC - Myeloablative conditioning, RIC - Reduced intensity conditioning, CMV - Cytomegalovirus, ECOG - Eastern Cooperative Oncology Group Performance Status

Table S6: Subgroup analysis for ECOG, NRM for CORE and ECOG combined

	CORE HCT[1]					ECOG Subgroup[1]			
	Pat. (%)	HR (95% CI)	*p* (%)	2-y (%)		Pat. (%)	HR (95% CI)	*p* (%)	2-y (%)
Total			<0.001		Total			<0.001	
CORE low	41	reference		10	ECOG 1+2	81	reference		15
CORE intermediate	51	2.43 (1.7-3.5)	<0.001	22	ECOG 3+4	11	2.96 (2.1-4.3)	<0.001	38
CORE high	8	6.09 (3.8-9.7)	<0.001	46	*missing data*	8			

[1]For the training, first and second validation with *n*=1025

Table 6 (continued):

	CORE and ECOG combined[2]					
	ECOG 1+2			ECOG 3+4		
	HR (95% CI)	*p* (%)	2-y (%)	HR (95% CI)	*p* (%)	2-y (%)
Total		<0.001			<0.001	
CORE low	reference		9	2.67 (0.6-11.7)	0.19	20
CORE intermediate	2.12 (1.4-3.2)	<0.001	18	4.96 (3-8.2)	<0.001	36
CORE high	6.21 (3.5-11.1)	<0.001	43	7.56 (3.7-15.6)	<0.001	48

[2]For the training, first and second validation with *n*=937 (minus missing data)

Figures

Figure S1: 2-Year NRM for CORE score for the first validation cohort

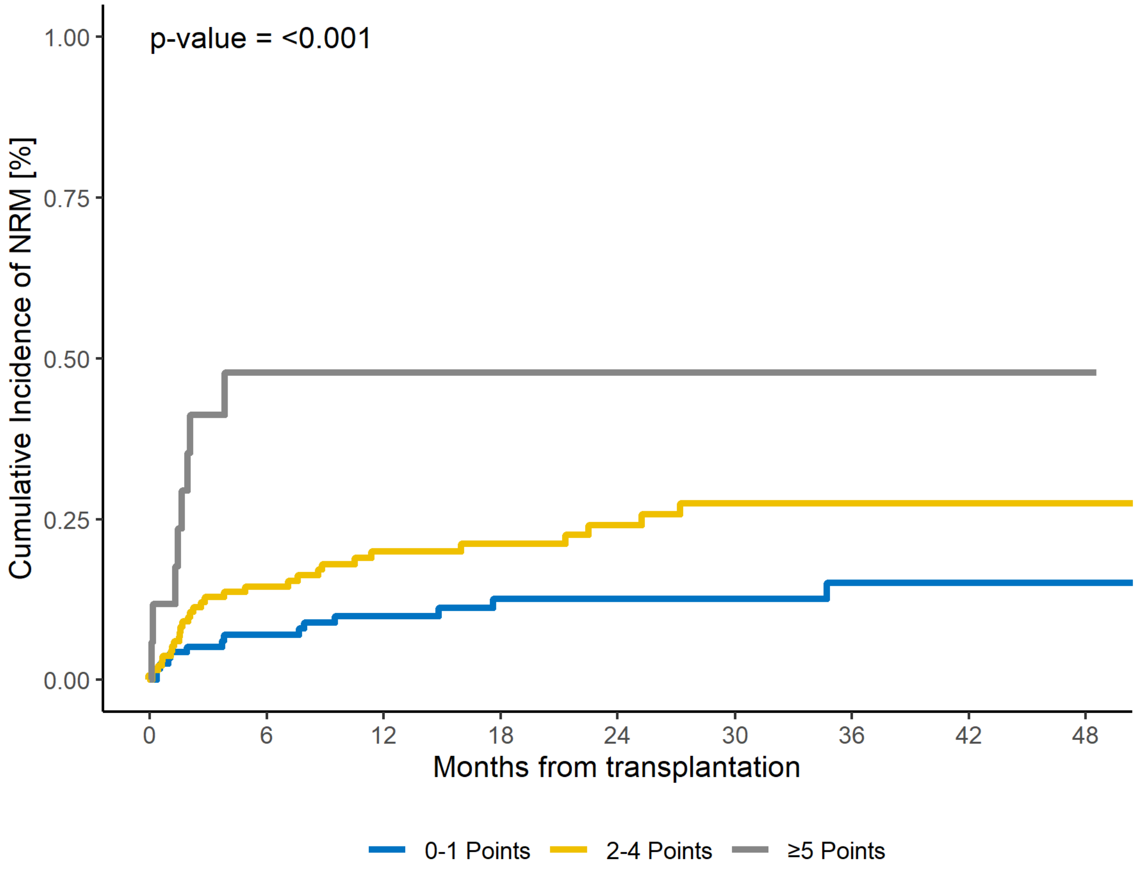

Figure S2: 2-Year OS for CORE score for the first validation cohort

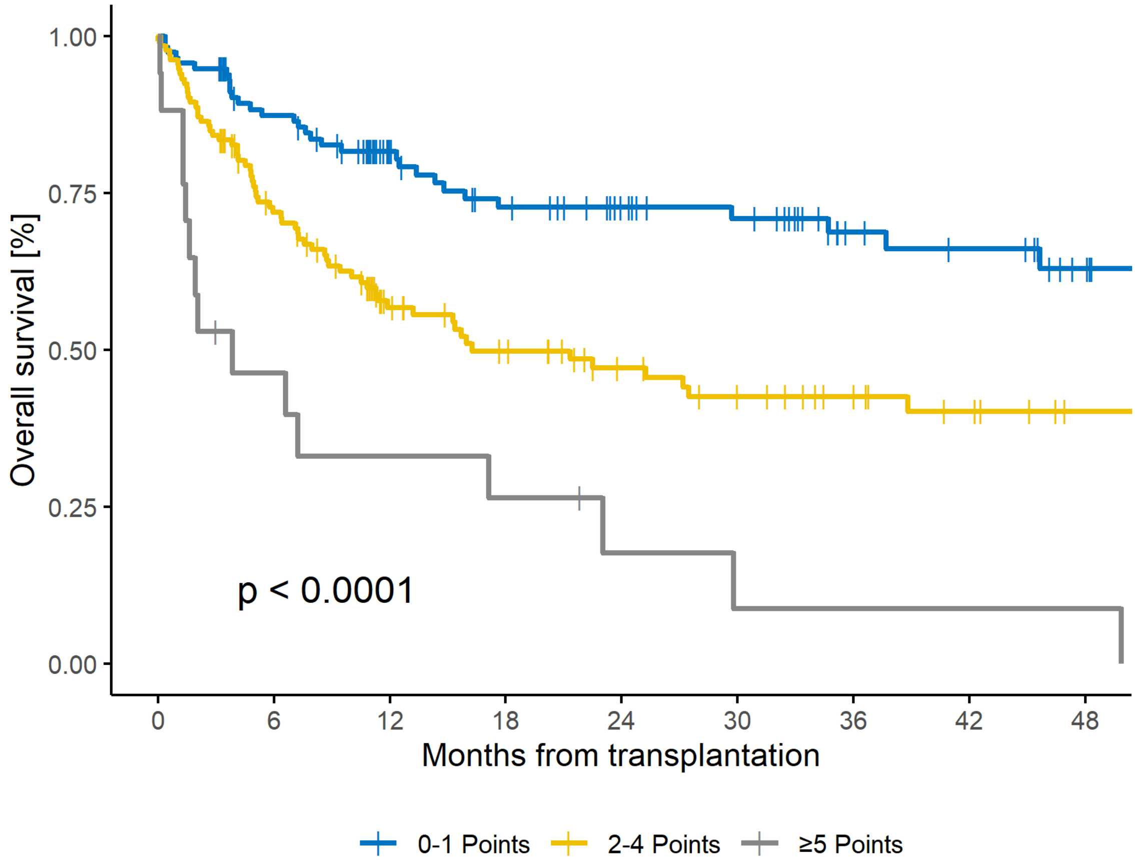

Figure S3: 2-Year NRM for CORE score for the second validation cohort

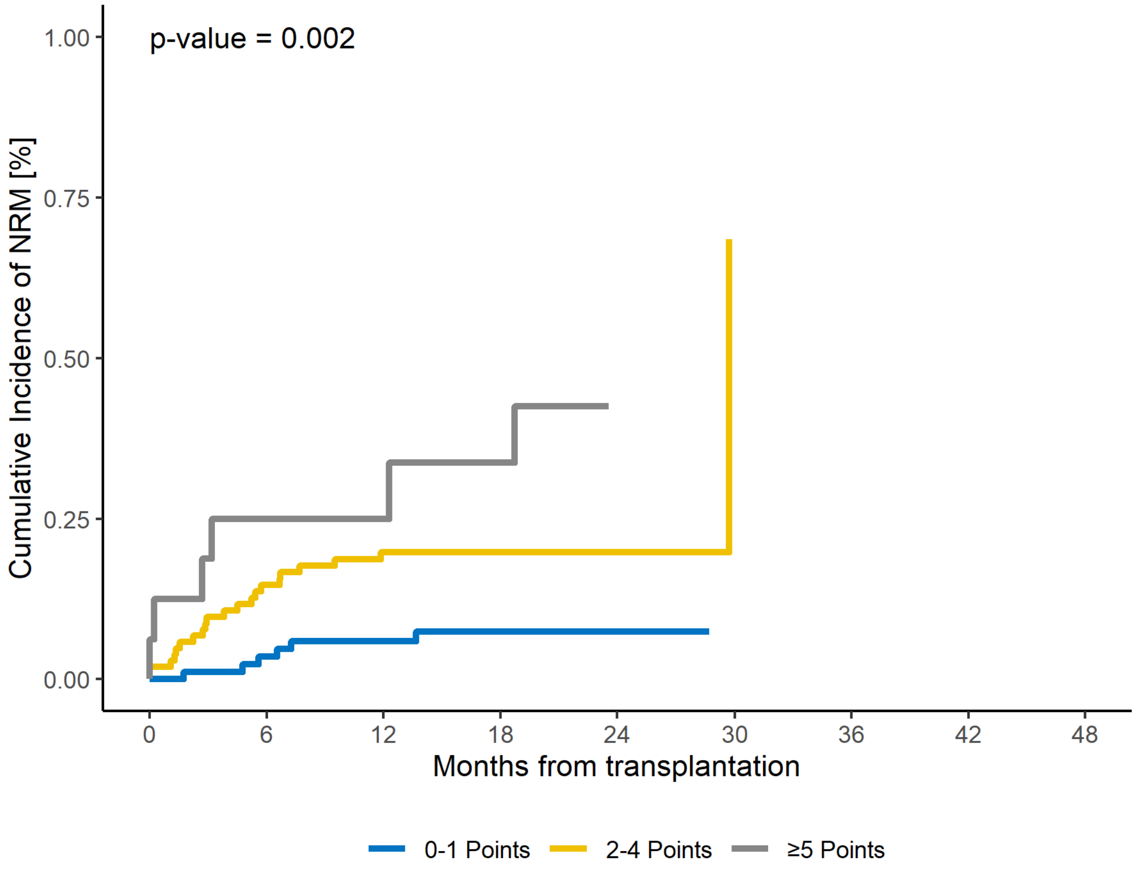

Figure S4: 2-Year OS for CORE score for the second validation cohort

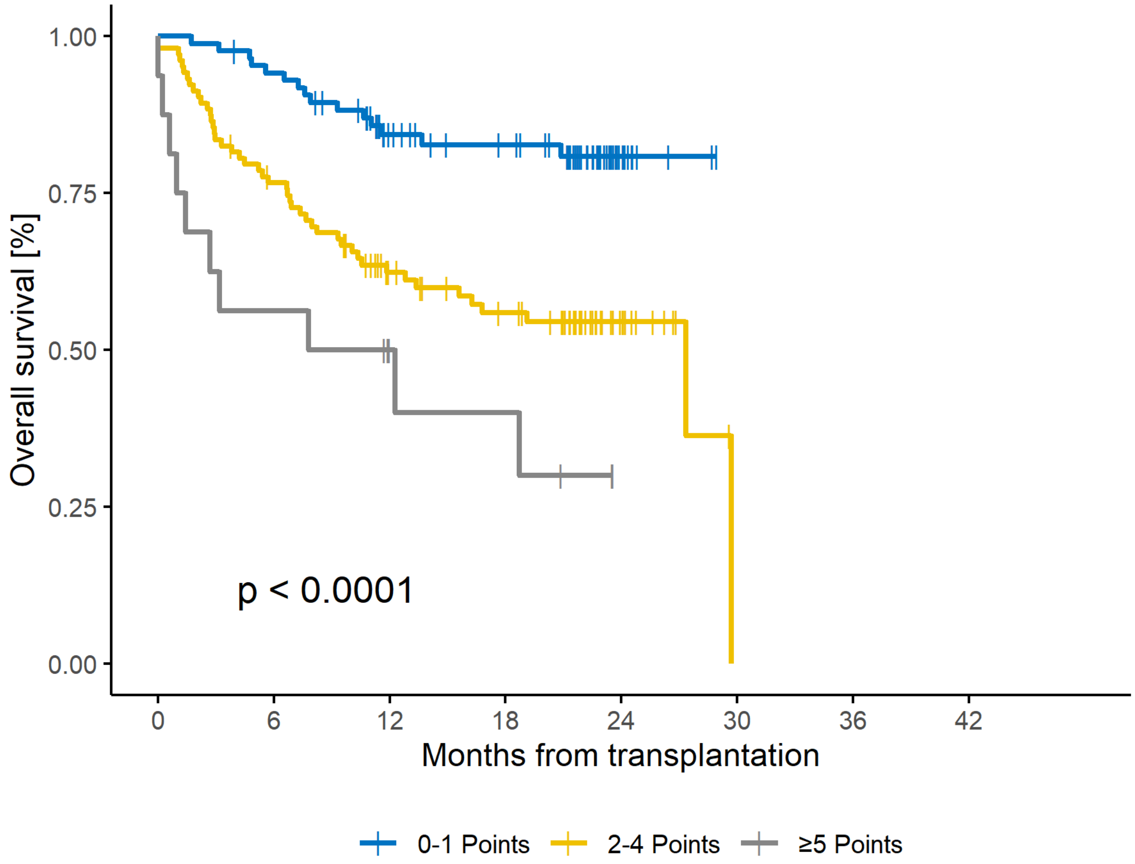

Figure S5: 2-year NRM for subgroup MAC

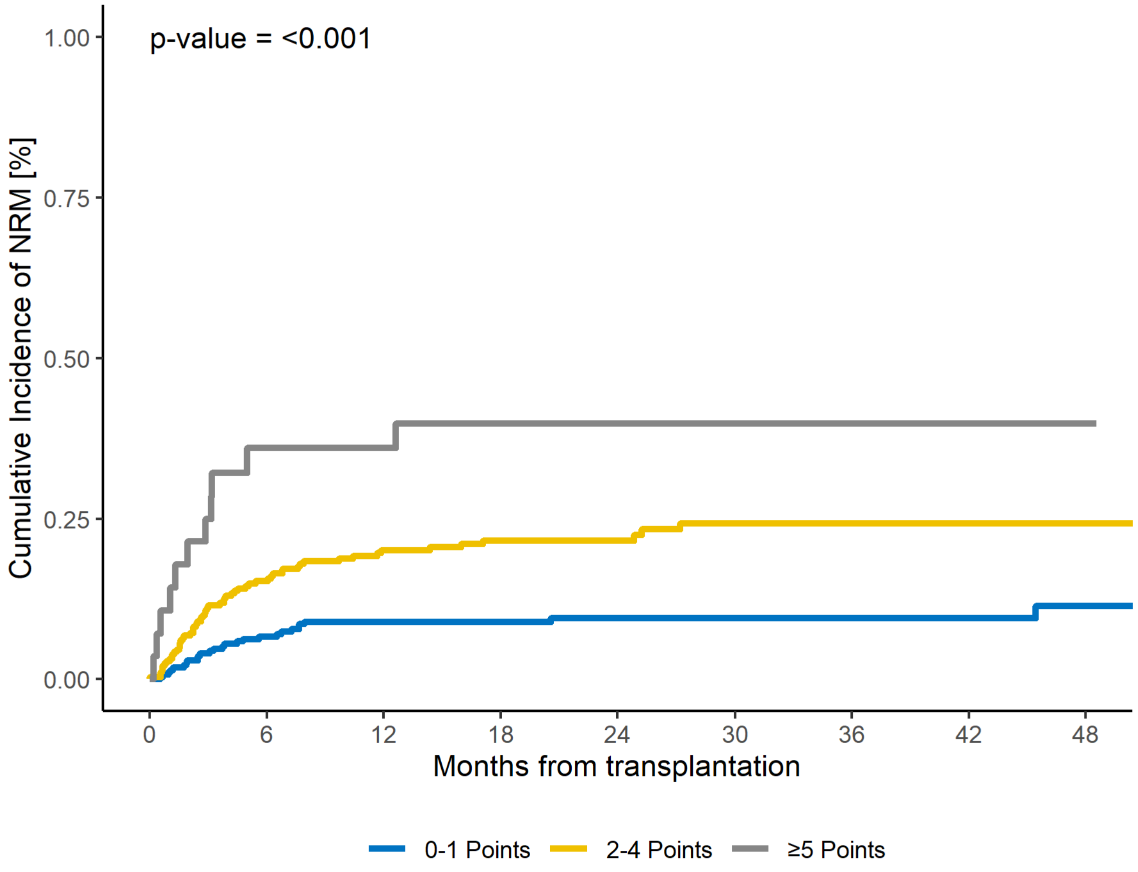

Figure S6: 2-year NRM for subgroup RIC

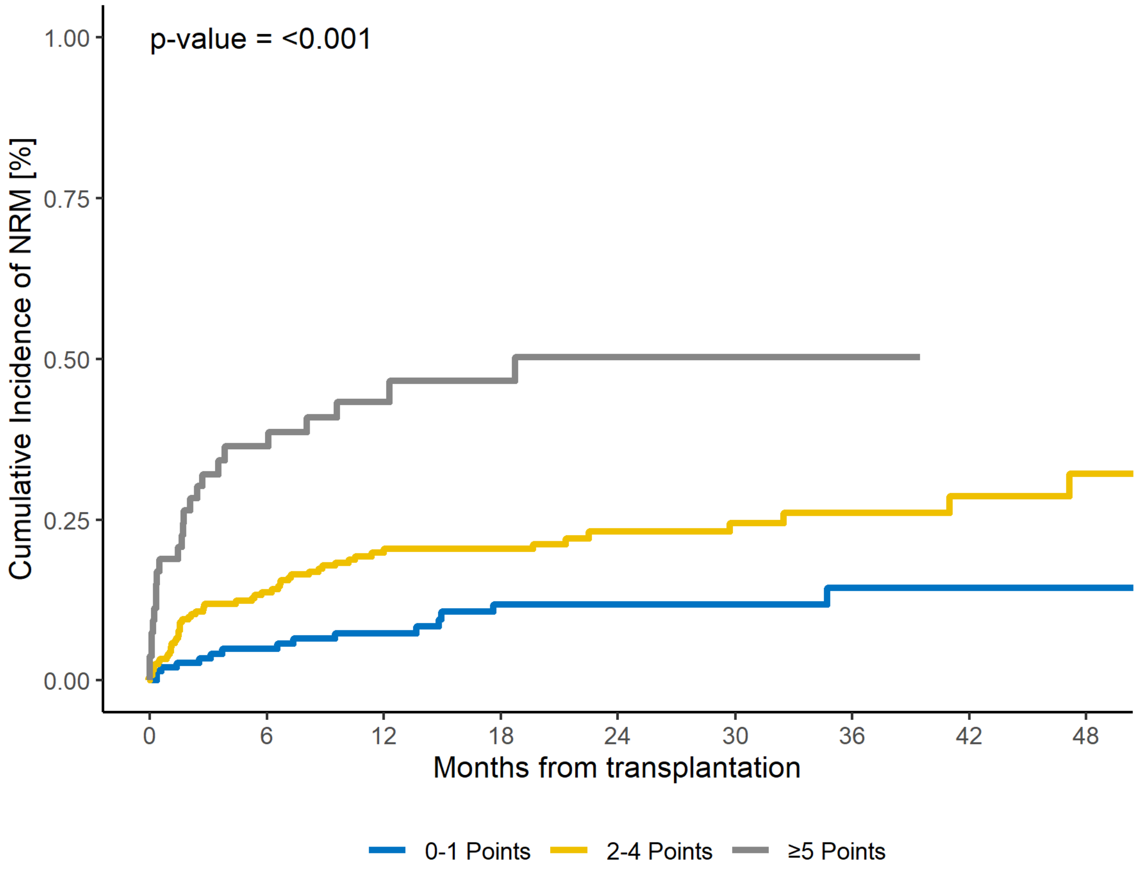

Figure S7: 2-year OS for subgroup MAC

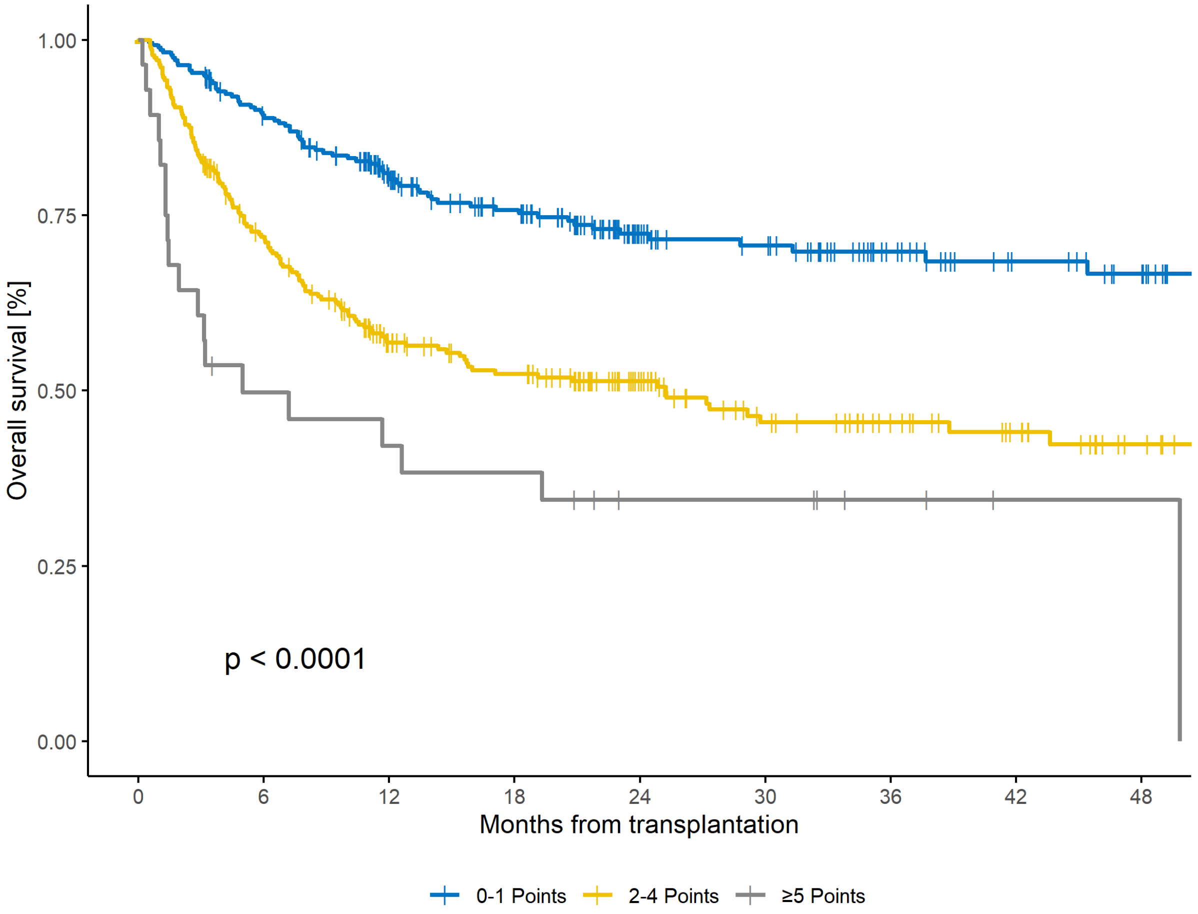

Figure S8: 2-year OS for subgroup RIC

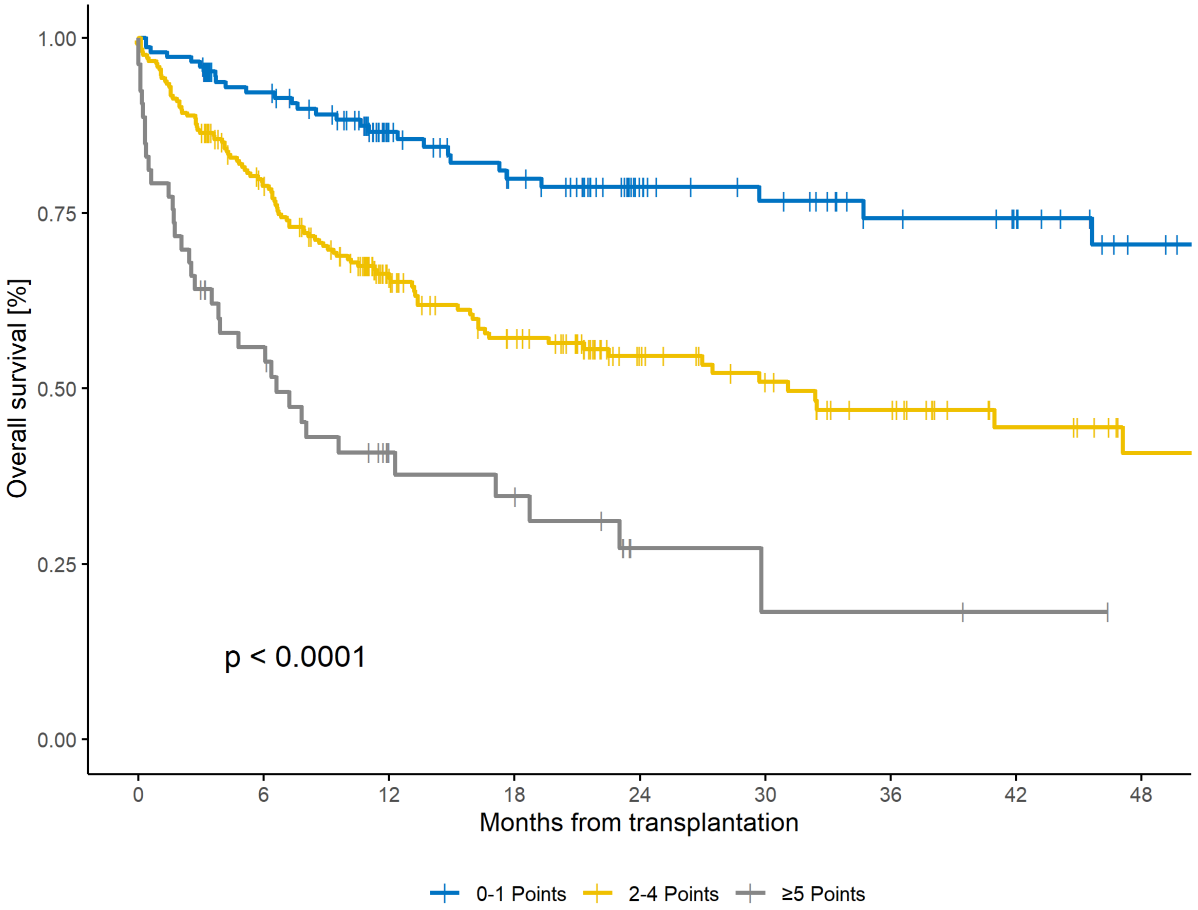

Figure S9: 2-year NRM of CORE score and ECOG status combined

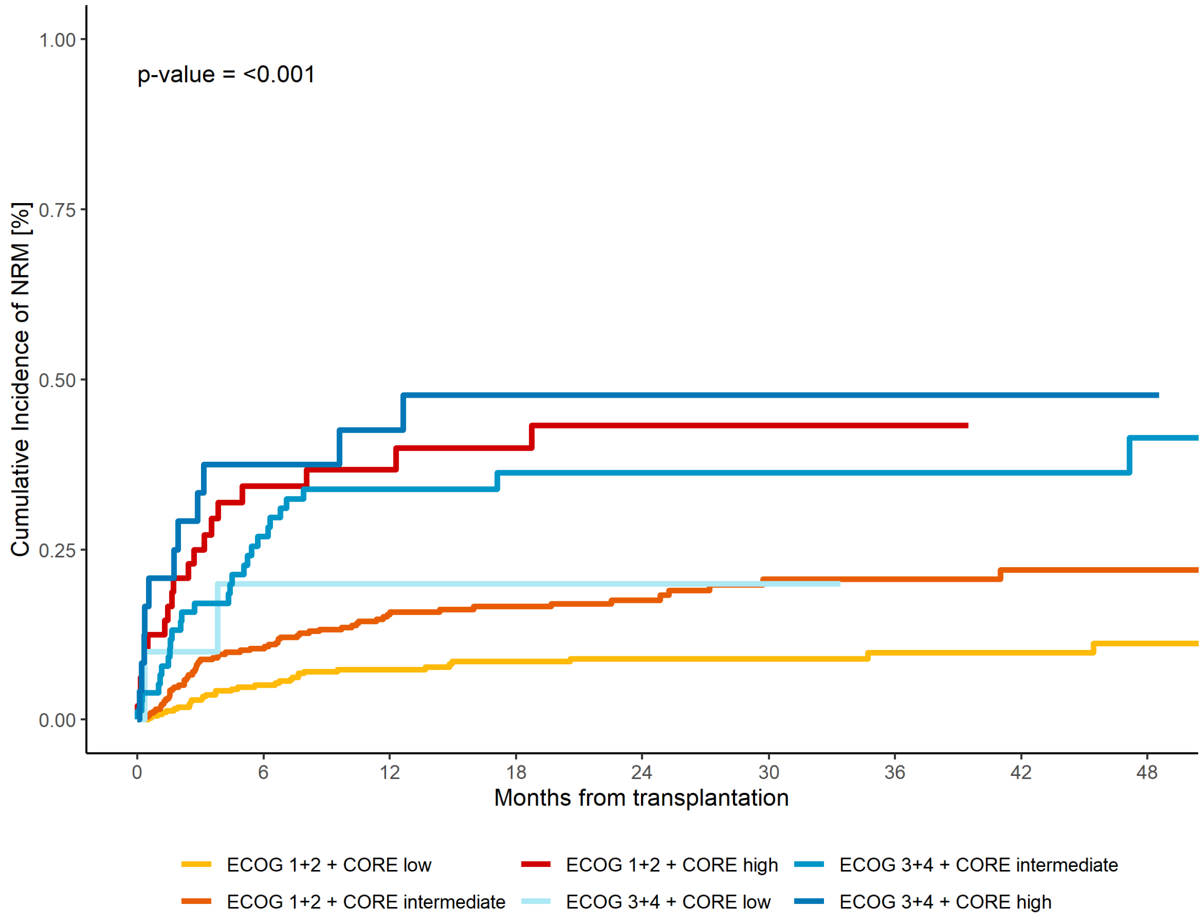

Supplement: Supplementary file 1 [file cancers-16-00515-s001.zip › cancers-2820449-supplementary.pdf]
